# Looking back into the Hepatitis C Virus epidemic dynamics from Unnao, India through phylogenetic approach

**DOI:** 10.1371/journal.pone.0317705

**Published:** 2025-01-16

**Authors:** Ajit Patil, Pallavi Vidhate, Sandip Patil, Amrita Rao, Swarali Kurle, Samiran Panda

**Affiliations:** 1 HIV Drug Resistance Laboratory—ICMR—National Institute of Translational Virology and AIDS Research, Pune, Maharashtra, India; 2 Division of Microbiology- ICMR—National Institute of Translational Virology and AIDS Research, Pune, Maharashtra, India; 3 Division of Clinical Sciences—ICMR—National Institute of Translational Virology and AIDS Research, Pune, Maharashtra, India; 4 Indian Council of Medical Research, New Delhi, India; Nazarbayev University School of Medicine, PAKISTAN

## Abstract

Viral hepatitis is a major public health challenge. Hepatitis C Virus (HCV) infection causes the progressive liver damage. A surprisingly high number of individuals tested positive for HCV infection during the Unnao Human Immunodeficiency Virus (HIV) outbreak investigation in 2017–2018 (more than 90% of the people living with HIV were from the Premganj township and Chakmeerapur village of the district in the northern State of Uttar Pradesh). This particular outbreak was attributed to the unsafe use of syringe & needles while seeking treatment, rendering it as an iatrogenic transmission. Earlier investigation towards, phylogenetic characterization revealed the shared ancestry for HCV sequences from the reported outbreak. In this investigation using the HCV sequences reported earlier (n = 67) we have analyzed the transmission linkages and evolutionary dynamics of this HCV outbreak using phylogenetic methods. In current analysis, time-scaled phylogenies indicated that most clusters initiated during 2015–2016. Transmission dynamics highlighted that the outbreak was experiencing an exponential phase during 2016–2017, where reproductive number was observed to be beyond documented values for HCV. Phylogeography revealed that source of the virus was Chakmeerapur and the effective population of Premganj was double the size of that in Chakmeerapur. In summary, we provide the snapshot of epidemic dynamics for HCV outbreak attributed to iatrogenic transmission.

## Introduction

According to the Global hepatitis report 2024, India reported 55, 00,000 total HCV infections in the year 2022. The population most at risk of HCV involves people who inject drugs, men having sex with men (MSM) and people who are prisoners. Moreover, unsafe medical injections are reported to be responsible for 13.8% of new HCV infections globally [1]. HCV is transmitted relatively more easily than HIV via intravenous injections [2]. HCV prevalence for India is estimated to be between 0.44 to 0.85% [3]. During 2017–2018, the Unnao district in the northern State of Uttar Pradesh, India, witnessed a high prevalence of HCV in the local community, where out of 158 individuals 98 (62.025%) were reported to be HCV seroreactive. Further investigations attributed this high level of prevalence within the community to the possibility of an iatrogenic transmission [4]. Molecular surveillance remains critical for timely redressal of such epidemics and more so in cases of localized outbreaks having a high incidence of viral infections [5]. Viral genetic similarity has been very well utilized to address the viral transmission networks leading to the inference of the linked transmission events [6, 7]. In addition to identifying linked transmission events, molecular sequences could be utilized to generate insights into the epidemic dynamics [8–11].

In the current investigation, we have summarized the transmission dynamics of the HCV epidemic reported from Unnao, India. Our analysis is based on the HCV NS5B sequences obtained during the Human Immunodeficiency Virus– 1 (HIV-1) and HCV dual outbreak investigation from Unnao, India [12]. Unnao investigation was initiated to address the increased detection of HIV among attendees at the integrated counselling and testing centre (ICTC) located in the district hospital. The investigation included ‘cases’ of individuals detected HIV sero-reactive from a defined geographical area during the study period either through ICTC or local health camps, while ‘controls’ were those who were detected as HIV sero-non-reactive during the same period and from the same sites. The striking feature of this case-control study was detecting HCV-antibody in high proportions in both cases and controls. Another key finding of this investigation was that none of the participants reported injecting drug use for recreational or non-medicinal purposes. However, the investigation revealed that most participants were exposed to intravenous or intramuscular injections while seeking healthcare [4].

Genetic characterization of the HCV sequences in earlier investigations highlighted the presence of genotype 3a; all were monophyletic. Moreover, the time to most recent common ancestor (TMRCA) of these sequences was inferred to be 2012 [12]. Likewise, the insights into the phylogenetic and evolutionary aspects of the HIV-1 sequences representing the same outbreak indicated the monophyletic clustering of HIV-1 pol gene region sequences and their most recent common ancestor to be close to 2012. In addition, HIV-1 transmission cluster reconstruction and phylodynamics aspects could elaborate upon the possible transmission links along with the trajectory of the epidemic growth in the study population. In the case of HIV transmissions, Bayesian demographic reconstructions could highlight the increased transmissions between 2012 and 2014 [13].

Even though the detected HCV prevalence was very high during the aforementioned outbreak investigation, key outbreak parameters like transmission clusters, epidemic dynamics, and viral migration patterns at the population level were not addressed. In this investigation, using HCV NS5B region sequences, we have elaborated upon the possible transmission linkages and HCV outbreak epidemic trajectory from Unnao.

## Materials and methods

### Dataset

In the current study, ancillary analysis was performed using the HCV sequences MW675899-MW675965 (n = 67) published earlier [12], under the investigation approved by the Institutional Ethics Committee (Ethical approval reference: NARI/SP/17-18/336 dated 29th May 2018) of the ICMR- National Institute of Translational Virology and AIDS Research (ICMR-NITVAR; erstwhile ICMR- National AIDS Research Institute-ICMR-NARI). These sequences were retrieved from the Los Alamos HCV sequence database (https://hcv.lanl.gov/content/sequence/HCV) and used for further analysis. The demographic details available were accessed in July 2024 and used for further interpretations.

### Genetic distance calculations and transmission network construction

Genetic distance calculations were performed using MEGA v6.0. Briefly, the sequences were aligned using MAFFT v7.4.50. This sequence alignment was submitted to MEGA, and the pairwise genetic distance calculations were performed using the Tamura-Nei model. The distance matrix file generated, was used to create a heatmap using ClustVis, a web tool for visualizing and clustering multivariate data [14].

To estimate the genetic distance thresholds for transmission network construction, we employed two methods: 1) AUTO-TUNE, and 2) Empirical Phylogeny Informed Cluster Tool (EPI-ClusT), a graphical user interface python programme for calculating optimum genetic distance threshold. The transmission network construction was carried out with the help of Microbe Trace [15] using a genetic distance threshold of 0.006, as estimated in earlier analysis. The sequence metadata available was provided as the node list for depiction purposes.

### Time scaled phylogeny

Bayesian maximum clade credibility (MCC) tree was constructed using BEAST v1.10.4 [16, 17]. Briefly, the 67 HCV NS5B sequences under consideration were used for time-scaled MCC tree construction. Since these sequences were homochromous, we employed the HKY+G substitution model with a strict molecular clock. The Bayesian Skyline was used as the tree prior. As for these sequences, the time to the most recent ancestor (TMRCA) was calculated earlier [12]; we used this information prior to setting the tree height using log-normal distribution (M = 6.0, S = 0.06) and setting the mean in real space. Simulations were performed for 500 million states, with a sampling of every 50,000 states. We employed the BEAGLE computation library during BEAST simulations [18]. The BEAST analysis convergence was further assessed with the help of Tracer v1.7.1 [19] with 10% burn-in. The final annotated MCC tree was generated with the help of Tree Annotator v 1.10.4 with 10% burn-in. The tree was visualized with the help of Fig Tree v 1.4.4.

### Outbreak phylodynamics and viral dissemination

We implemented the BEAST v2.6.7 for the Bayesian skyline and Birth-Death Skyline (BDSKY) Contemporary analysis [20, 21]. We used the HKY+G substitution model with a strict molecular clock for both analyses. Tree height was set using log-normal distribution (M = 6.0, S = 0.06) and setting the mean in real space as the TMRCA (2012.0) of this dataset was known [12]. For BDSKY analysis, we used the following priors, log-normal distribution (M = 0, S = 1.0) for reproductive number R, which places the median at 1 with a majority weight below 5.18. For becoming un-infectious rate, log-normal distribution (M = 0, S = 1.25) was used, setting the un-infectious rate quantile between 31 days and 11.5 years. Sampling probability (rho) was set with beta distribution (Alpha = 1.0, Beta = 999), reflecting only the fraction of outbreak-related samples collected. The epidemic’s origin was set using log-normal distribution (M = 2.5, S = 0.25), which sets the origin before the calculated TMRCA for these viruses. Simulations were performed for 300 million chains with sampling at every 30000 states. Convergence of the BEAST analysis was assessed using Tracer v1.7.1 with 10% burn-in. Bayesian Skyline graphs were generated with the help of Tracer v1.7.1. The BDSKY plot was generated in RStudio v 1.3.959 integrated development environment (IDE) using the R-functions of “bdskytools” package.

Further, to infer the viral dissemination between two localities in the Unnao district, namely Chakmeerapur and Premganj, we employed the Marginal Approximation of the Structured COalescenT (MASCOT) analysis [22] implemented in BEAST 2. The HKY+G substitution model was used with a strict molecular clock for this analysis. Tree height was set using the log-normal prior (M = 6.0, S = 0.06) with mean in real space, setting the TMRCA to 2012.0. The mascot was set as the tree prior, and the sampling locations were assigned to each tip (Chakmeerapur or Premganj). As every sample might not have been sampled from the respective locations, the prior for effective population constant (Ne) was set using log-normal distribution (M = 0, S = 1.0). The simulations were performed with 10 million steps and sampling every 1000 steps. The annotated MCC tree was generated with the help of Tree Annotator v2.6.7. The final annotated tree was visualized using Fig Tree v1.4.4

## Results

### Sequences demography

Of the 67 analyzed sequences, 38 (56.71%) were from the residents of Premganj, 28 (41.79%) from Chakmeerapur, and 1 (1.49%) from Kirvidyapur. Most infected individuals were female (n = 38, 56.71%), while 28 (41.79%) were male. Overall, there were 23 sequences which belonged to the individuals infected with HCV and HIV. There were 44 sequences, which were from individuals infected with HCV only. Out of 23 sequences from individuals co-infected with HCV and HIV, the majority (n = 16) were from females, while 7 were from males. Out of 44 sequences from individuals infected only with HCV, males and females contributed equally, 22 each. Most individuals were married (n = 60, 89.5%), while 6 (8.9%) were unmarried, and one was a divorcee. None of the unmarried individuals reported having sex with a casual sex partner or visiting a female sex worker. However, from married respondents, only 2 reported having sex outside marriage (with casual sex partner or female sex worker). Regarding injections administered for medical purposes, 48 (71.64%) individuals reported having intravenous or intramuscular injections in the last five years, and 41 (61.19%) reported receiving intravenous and intramuscular injections in the previous five years. As reported earlier most of these individuals were exposed to the used syringes and needles while seeking medical treatment, most often from local treatment provider [4]

### Sequence relatedness and transmission networks

We calculated the pairwise genetic distance between these sequences to infer upon their relatedness to each other. The mean genetic distance calculated for these 67 sequences was 0.0097. Further as shown (**S1 Fig**) in the heatmap generated using the pairwise genetic distance matrix, the sequence relatedness can be highlighted. The maximum pairwise genetic distance observed was 0.032, while the minimum was 0.00. Moreover, as depicted in the heat map, subsets of sequences had zero genetic distance. Among these subsets of sequences having zero genetic distance, the largest cluster contained 20 sequences. Out of these 20 sequences having zero genetic distance, 15 were from Premganj, and five were from Chakmeerapur. Fourteen of these 20 sequences were from individuals having HCV infection, and the remaining 6 were co-infected with HCV and HIV-1.

Selecting an optimum distance threshold for inferring epidemiologically linked transmissions is crucial for analyzing the structures of inferred networks. For this reason and to avoid any biases, we applied Empirical Phylogeny Informed Cluster Tool (EPI-ClusT) and recently developed general heuristic scoring approach AUTO-TUNE for establishing the best threshold for our sequence dataset. Using methods the optimum distance threshold calculated for our dataset was 0.006.

**Fig 1:**
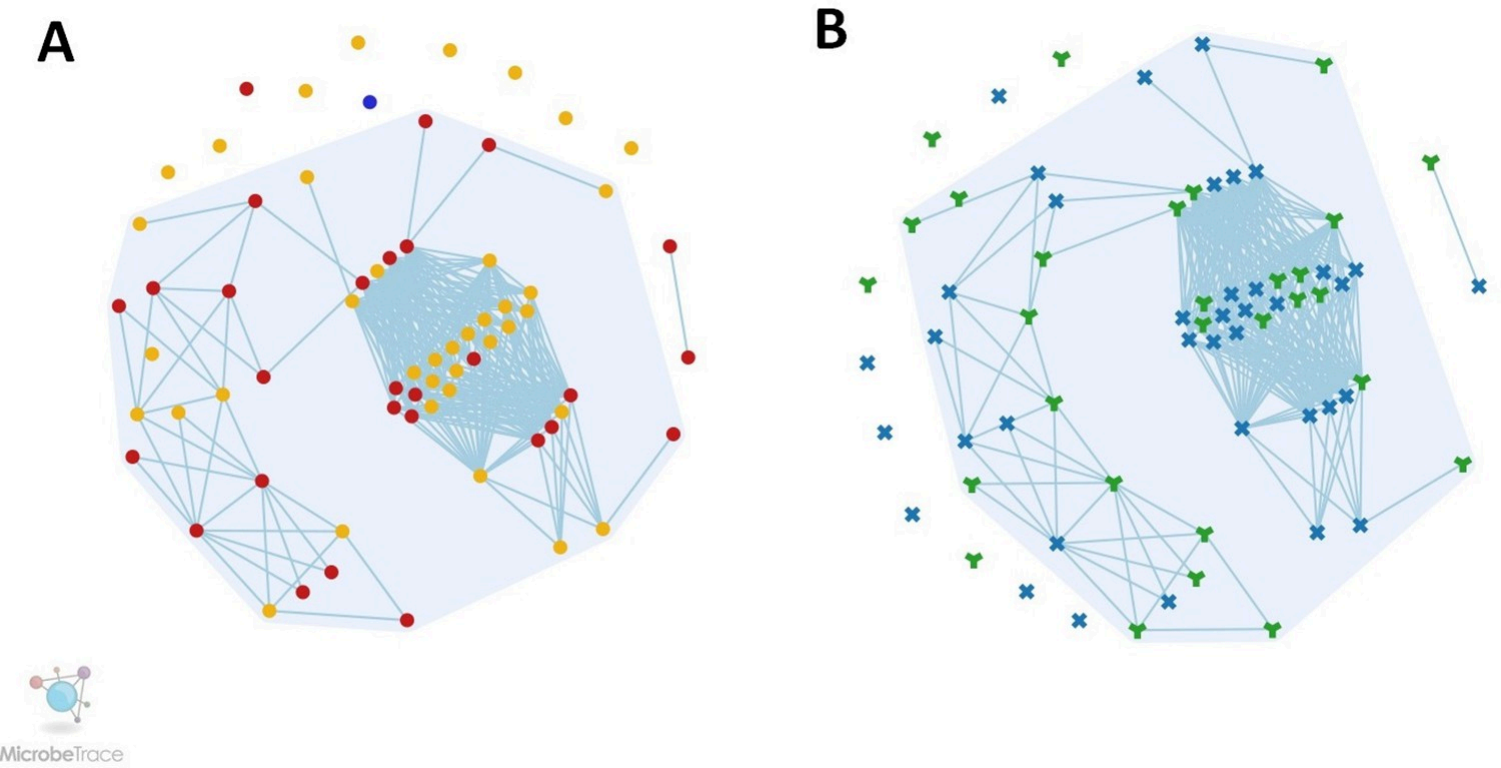
**A)** Transmission network representation. The yellow colored circles represents the premganj, while red is for chakmeerapur and blue indicates the Krividyapur. **B)** Transmission network representation with respect to sex of the individual participants. The blue colored multiplication sign denotes the females while green colored “Y” sign indicates the males.

Using the genetic distance threshold of 0.006, we constructed the transmission network with the help of MicrobeTrace. Unnao sequences became segregated into two clusters. The largest cluster had 54 sequences linked, while the other cluster contained only two sequences. The remaining 11 sequences were observed to be singletons. The largest cluster constituting 54 nodes had mean genetic distance of 0.0024, and the median age of the individuals in this cluster was 44.5 year. This cluster contained 23 males and 31 females. As shown in figures (**Fig 1A and 1B**), most individuals within this cluster were from Premganj (n = 29), while remaining (n = 25) were from Chakmeerapur. Two individuals in the other cluster (one male, and one female) hailed from Chakmeerapur. The mean genetic distance for this cluster was 0.0022.

### Time-scaled phylogeny

**Fig 2:**
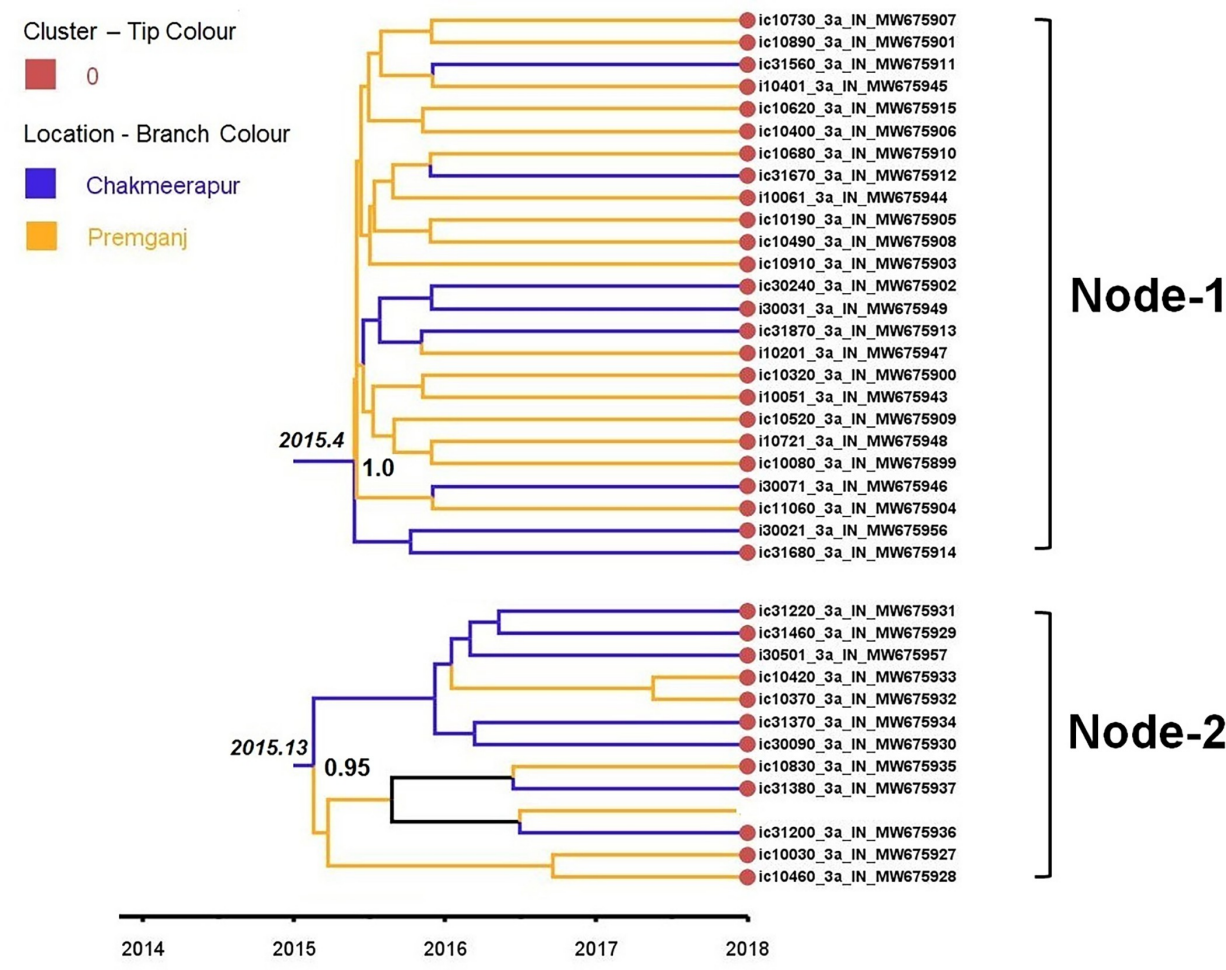
Nodes form the time-scaled MCC tree highlighting the TMRCA of the individual transmission clusters. As depicted in the legend, the blue colored branches represent the location chakmeerapur, while yellow branches indicate the location premganj. The red circles at the end of the branch tips highlight the specimens belonging to transmission cluster– 0.

The largest transmission cluster constituted 54 sequences. As these were observed to be the largest fraction of epidemiologically linked sequences representing this dataset, we examined the time scale resolution of these transmission events. As shown in the figure **(Fig 2)**, the nodes comprising the majority of the largest cluster (Cluster 0, n = 37) sequences had the TMRCA around 2015 (Node-1 and Node-2). The node at the top (Node-1) constituted 25 sequences representing cluster 0 and the TMRCA estimated for these sequences was 2015.4 (95% HPD: 2013.84–2016.87). Out of 25 sequences in Node-1, 17 were from Premganj, while the remaining eight were from Chakmeerapur. The 20 sequences with 0 genetic distance were part of this node. The TMRCA for the node at the bottom (Node -2) was observed to be 2015.13 (95%HPD: 2013.61–2016–67). Node-2 contained a total of 12 sequences representing cluster 0. Out of these 12, the majority (n = 7) were from Chakmeerapur, while the remaining five were from Premganj. Moreover, as shown in the S2 Fig, the other sequences inferred to be a part of cluster 0 (highlighted in yellow) had the TMRCA around 2015–2016. The cluster containing two sequences (highlighted in blue) only had the TMRCA of 2016.97 (95% HPD: 2015.91–2017.98). Overall, these observations highlight that all the inferred transmission clusters had their earliest common ancestor aged 2015–2016.

### Population dynamics

**Fig 3:**
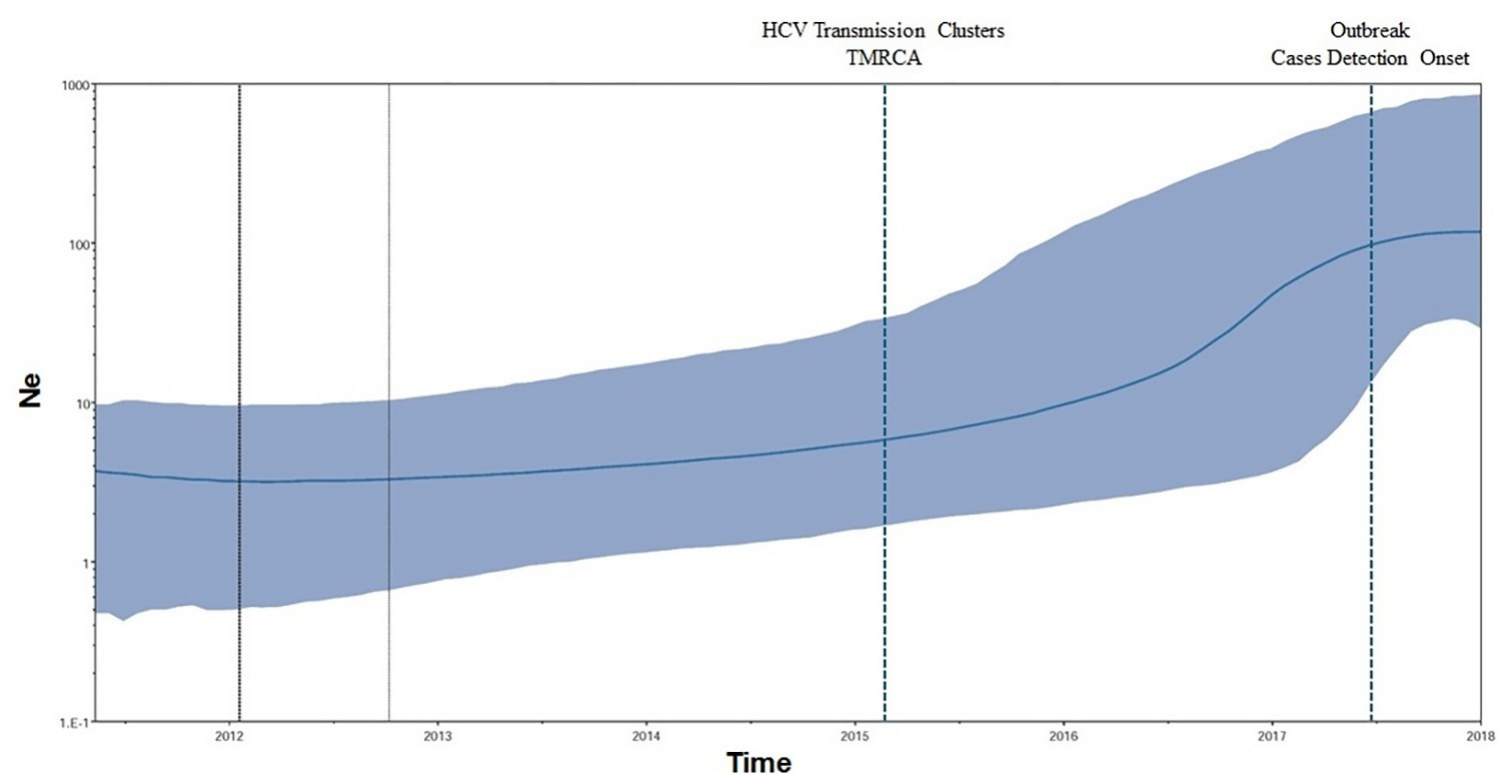
Bayesian skyline plot highlighting the effective population size (Ne) in log scale over the time. The solid line represents the median estimates while the gray-shaded area indicates the 95% HPD. Vertical black lines represent the estimated lower and median 95% HPD pertaining to time scale. Vertical dotted blue lines drawn represents the timelines of inferred TMRCA for transmission clusters and outbreak investigation start period respectively.

Phylodynamic analysis was undertaken to understand the spread of this viral strain in the population. The population level dynamics inferred through the usage of the skyline plot **(Fig 3)** highlighted that the effective population had already entered an exponential phase of growth starting from the middle of 2015. In between the start of the year 2017 till its middle, the infected population experienced a rise of almost 1 log. Thereafter, from the middle of 2017 till the onset of 2018 the effective population size did not observe any significant rise.

**Fig 4:**
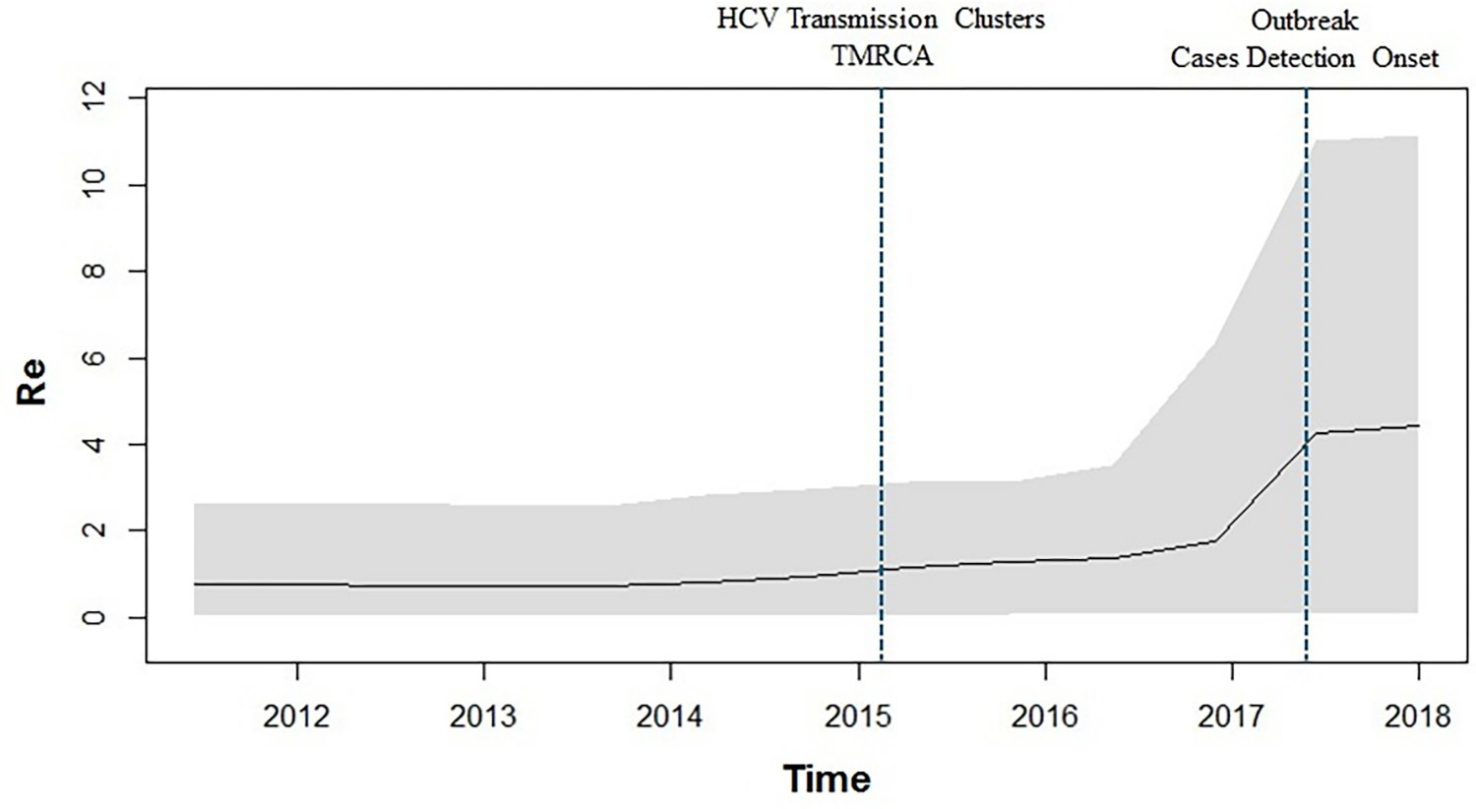
Birth death skyline plot demonstrating the estimates of median effective reproductive number (Re) over time. Vertical dotted blue lines drawn represents the timelines of inferred TMRCA for transmission clusters and outbreak investigation start period respectively.

The BDSKY analysis **(Fig 4)** revealed that the effective reproductive number (Re) for this viral strain was around 1 till the middle of 2014, starting from its TMRCA of 2012.0. Eventually, beginning in 2015 and till 2017, a gradual and upward trend emerged, where Re was close to 2 around 2017. Noticeably, from the start of 2017 till the middle of 2017, Re almost touched the value of 4. After that, from mid-2017 till 2018, Re was stationary.

### Dissemination within the locality

**Fig 5:**
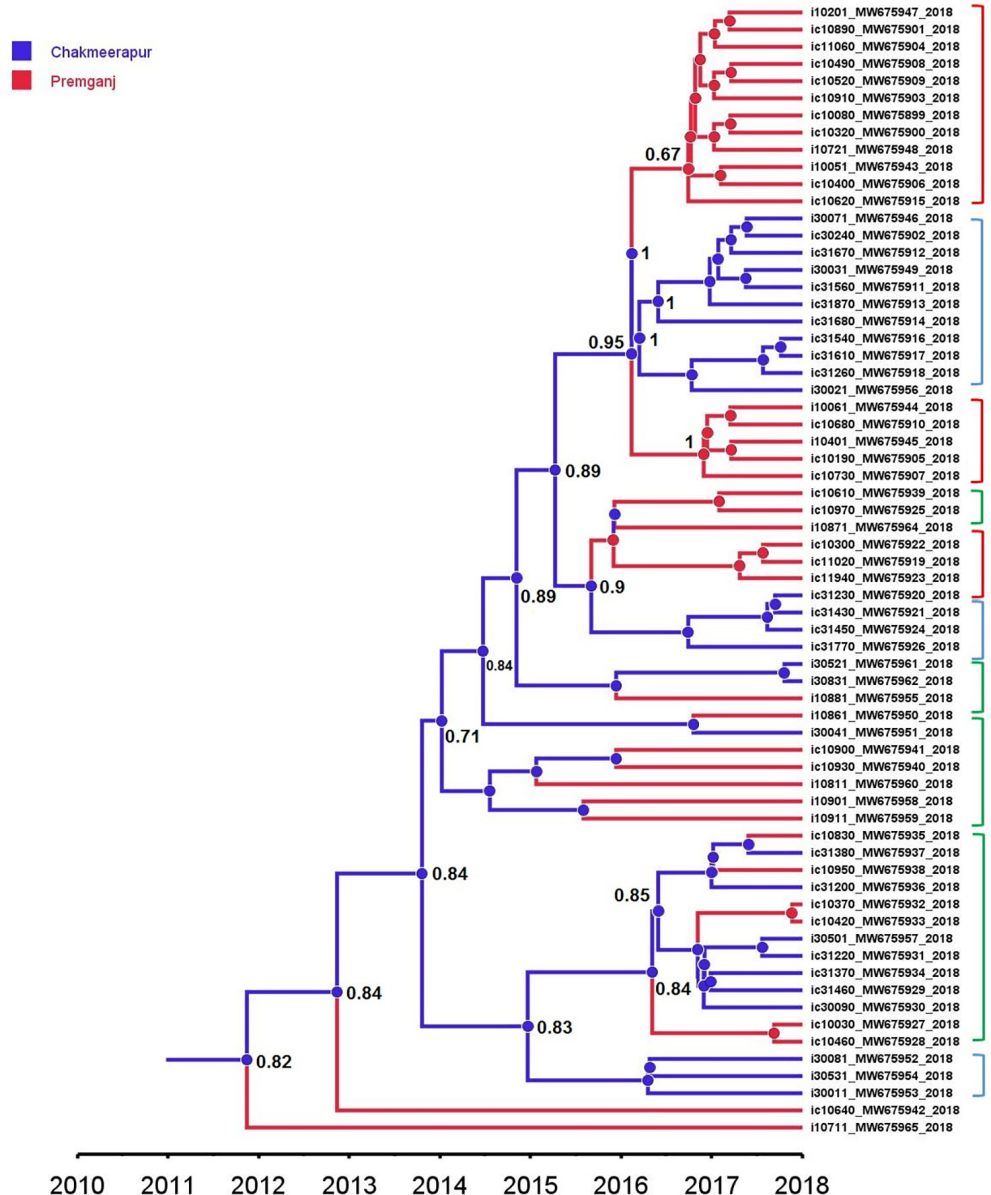
MCC tree obtained after the MASCOT analysis. The branches and node shape colors are set according to the locations. The blue color represents the chakmeerapur while red denotes the premganj. The figures near the nodes indicate the maximum location probability. The square brackets on the right side indicate the transmission events with respect to geographical locations inferred–where red brackets indicate the transmission events pertaining to premganj, blue brackets representing the transmission events within chakmeerapur while the green brackets indicates the viral migrations between premganj & chakmeerapur.

**Fig 6:**
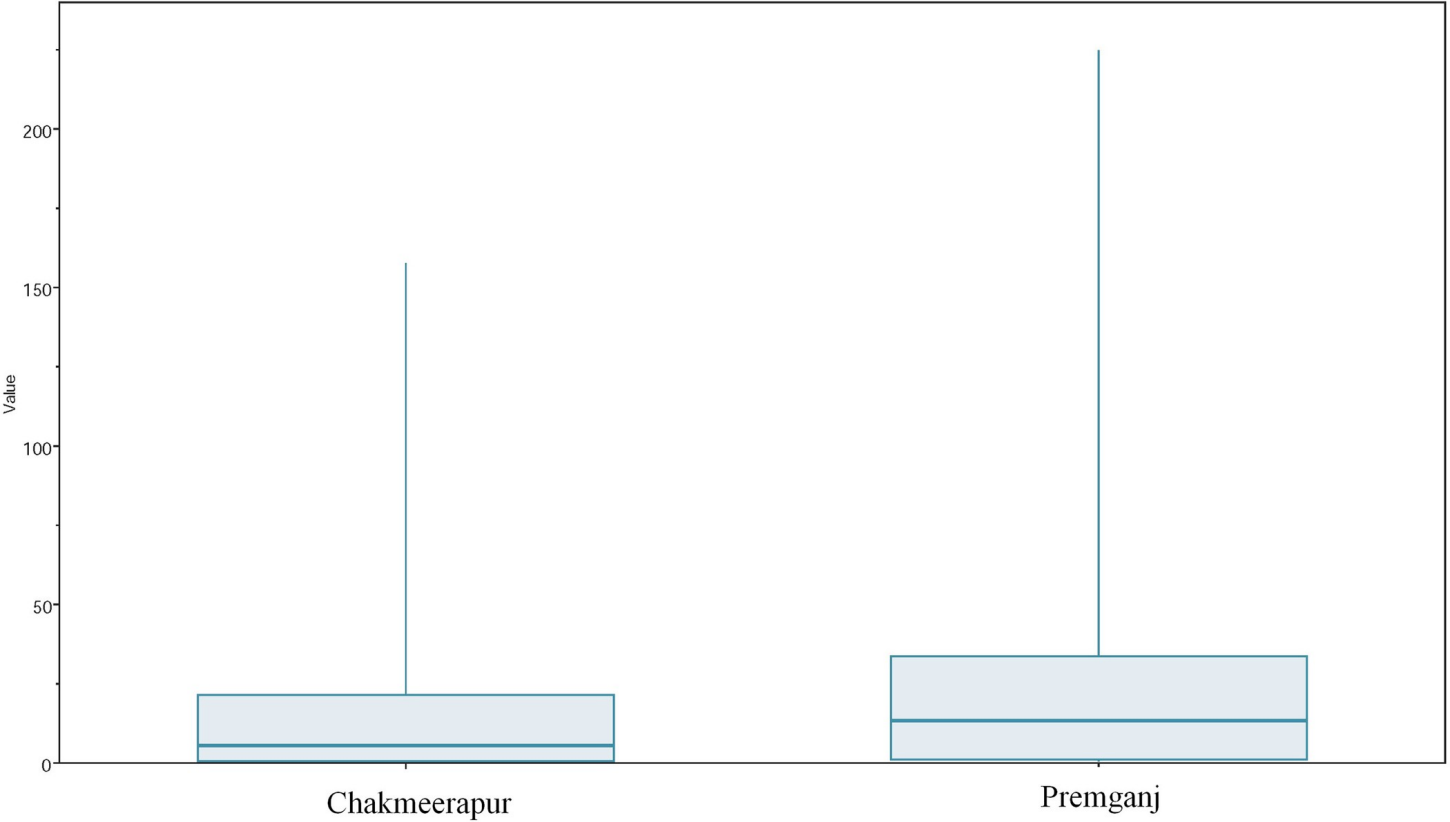
Graph representing the effective population size constants.

To infer the within-locality (Chakmeerapur and Premganj) transmissions and differential effective populations in Chakmeerapur and Premganj, respectively, we employed the Marginal Approximation of the Structured COalescenT (MASCOT) analysis. As shown in the MCC tree obtained after MASCOT analysis **(Fig 5)**, the most likely source of the virus was Chakmeerapur. As shown, the node at the base represents the location being inferred as Chakmeerapur with the maximum location probability of 0.82. It can be observed very well that moving ahead in time, the location inferred for majority of the nodes was Chakmeerapur with sufficient posterior probability support. In addition, there were sub-clades within the tree, which contained the nodes having their location inferred as Chakmeerapur (blue brackets) or Premganj (red brackets) and were observed to lead to viral transmission events within the same locality. However, there were clades representing mixed transmission events (green brackets). The effective population size within the Chakmeerapur and Premganj were observed to be different. Marginal posterior distribution graphs **(Fig 6)** highlighted that Premganj had almost double the effective population size than that of Chakmeerapur, where the mean Ne constant for Premganj was 13.349 (95% HPD: 1.07–33.63) and 5.52 (95% HPD: 0.61–21.49) for Chakmeerapur.

## Discussion

According to the Global hepatitis report 2024, the prevalence of hepatitis C in South-East Asia region in the year 2022 was 0.5% [1], and the HCV prevalence for India as estimated between 0.44 to 0.85% [3]. The Unnao HIV outbreak investigation unearthed an exceptionally high rate of HCV infection in the study participants. While 86% of HIV sero-reactive ‘cases’ had concomitant HCV-antibody, the more alarming finding was the presence of HCV-antibody in 56% of the ‘controls’ who were *not* HIV sero-reactive. [4]. In addition to these observations, the study by Mane et al, highlighted that the tree topology of NS5B sequence phylogenies indicated the monophyletic nature of HCV infection supporting the use of unsterile injection in health care seeking as the cause of these transmissions [12]. Viral sequences are critical to understanding viral spread, identifying outbreaks and inferring the outbreak dynamics. Moreover, these sequences can be used to identify the connection amongst cases, particularly in case of suspected outbreaks [5, 23, 24].

Sequence information can be utilized for the identification of linked transmission events, eventually leading to growing transmission clusters [25]. In case of HIV transmissions during Unnao outbreak, it was observed that the virus within the locality possessed the shared ancestry, and the transmission events were linked. Moreover, demographic reconstruction could highlight the timelines of the trajectory followed by these transmission events at the population level. These insights exemplified that this outbreak certainly had a period when the effective population experienced exponential growth, indicating the rapid transmission events [13]. Phylogenetic analysis of the HCV sequences in a follow-up study by Mane eta al could highlight the presence of a single HCV subtype having shared ancestry [12].

Leading on to earlier investigations [4, 12, 13] we sought to explore the probable HCV transmission linkages and outbreak dynamics. Our observations could highlight the noteworthy viral homogeneity indicated by the pairwise genetic distances among the study sequences. Even though the participants did not report any injecting drug use for recreational or non-medicinal purposes, the presence of transmission linkages in the majority of the study participants alluded to the spread of the virus having shared ancestry and reaffirming the common source of infections. Moreover, the exposure of the majority of the participants to the injections during healthcare seeking reconfirms the possibility of iatrogenic transmissions. Insights of the epidemic dynamics revealed the trajectory followed by these transmissions in time and space.

Uttar Pradesh State AIDS Control Society (UPSACS) governed ICTC facilities experienced increasing trends of HIV sero-reactive cases during 2015–2018 [4]. Surprisingly, the time-scaled phylogenies constructed for HCV sequences during this investigation highlighted the TMRCA of the transmission clusters to be 2015–2016. In addition, we could observe the extraordinary level of sequence homogeneity in certain clades, where, in some cases, the genetic distance between these sequences was observed to be 0.0. Further, the demographic reconstructions indicated that 2015 to 2017 was the period where rapid transmission events took place, and it further exploded during 2017–2018. These observations are in close corroboration with the trends of the HIV sero-reactive cases reported for a period of 2015–2018 by UPSACS and ICTC centers [4]. Although presently, the reproductive number for HCV is considered 1.2–2.9 [26], our BDSKY analysis revealed that during the outbreak-period, the reproductive number reached 4. Overall, these findings indicated a rapid and explosive spread of the circulating HCV strain from a single source.

Since most of the sequences sampled were from the Chakmeerapur and Premganj localities of the Unnao district, the obvious question was what could be a possible source of virus in these two locations? Chakmeerapur and Premganj are the localities within the Bangarmau block of Unnao district, Uttar Pradesh. These two localities are located on the opposite ends of the Bangarmau block with 5.0 km distance between them. We implemented the phylogeographic analysis MASCOT to understand the possible geographical source (Chakmeerapur or Premganj) of the virus and perpetual virus introductions within these localities. Chakmeerapur was estimated to be the plausible origin of spread of virus between Chakmeerapur and Premganj, perpetually these introductions were estimated to be more plausible from Chakmeerapur compared to Premganj. MASCOT could also highlight that as these introductions took place, the infected population in Premganj was more than in Chakmeerapur. These findings indicated that even though the transmission events initiated from Chakmeerapur the disease burden was more in Premganj.

Overall, our analysis highlighted that there were linked HCV transmission events during the Unnao outbreak. From the time-scaled phylogenies, it could be estimated that the actual outbreak was initiated sometime between 2015 and 2016. The most concerning factor, however, was the rapidity of the transmission events observed in the form of our reproductive number estimates. The inferred reproductive number for HCV in this outbreak was observed to be almost double the highest value for HCV.

In conclusion, these findings reveal the nature of the transmission linkages and the spatiotemporal dynamics of the HCV outbreak from Unnao, India. Phylogenetic and phylodynamic aspects of the viral spread into the community are critical for the contemporary assessments, forecasts and insights to control the future emergence. Moreover, the use of viral evolutionary information combined with available metadata about demographic characteristics has the potential to inform the public health systems for carving the necessary response system [23, 24]. Indeed, our observations provide critical information about the HCV outbreak from Unnao and a mini-roadmap on how public health systems in India can implement molecular surveillance strategies to investigate the key parameters of any ongoing or past outbreaks. Moreover, on the therapeutic front the takeaway message is to prioritize the implementation of safe injection practices along with enforcement of strong regulatory frameworks for ensuring safety.

## Supporting information

S1 Fig Heatmap representation of the pairwise genetic distances between HCV NS5b sequences. The color scale on the right depicts the color schemes applied for particular genetic distance thresholds.
(TIF)

S2 Fig Bayesian time-scaled MCC tree. The legend on the left identifies the colors used to represent the individual locations. The figures at individual nodes represents the posterior probability.
(TIF)
