# Supplementary figures and images for: Looking back into the Hepatitis C Virus epidemic dynamics from Unnao, India through phylogenetic approach
Source: PLoS One. 2025 Jan 16;20(1):e0317705. doi: 10.1371/journal.pone.0317705 (PMC11737721; doi:10.1371/journal.pone.0317705)

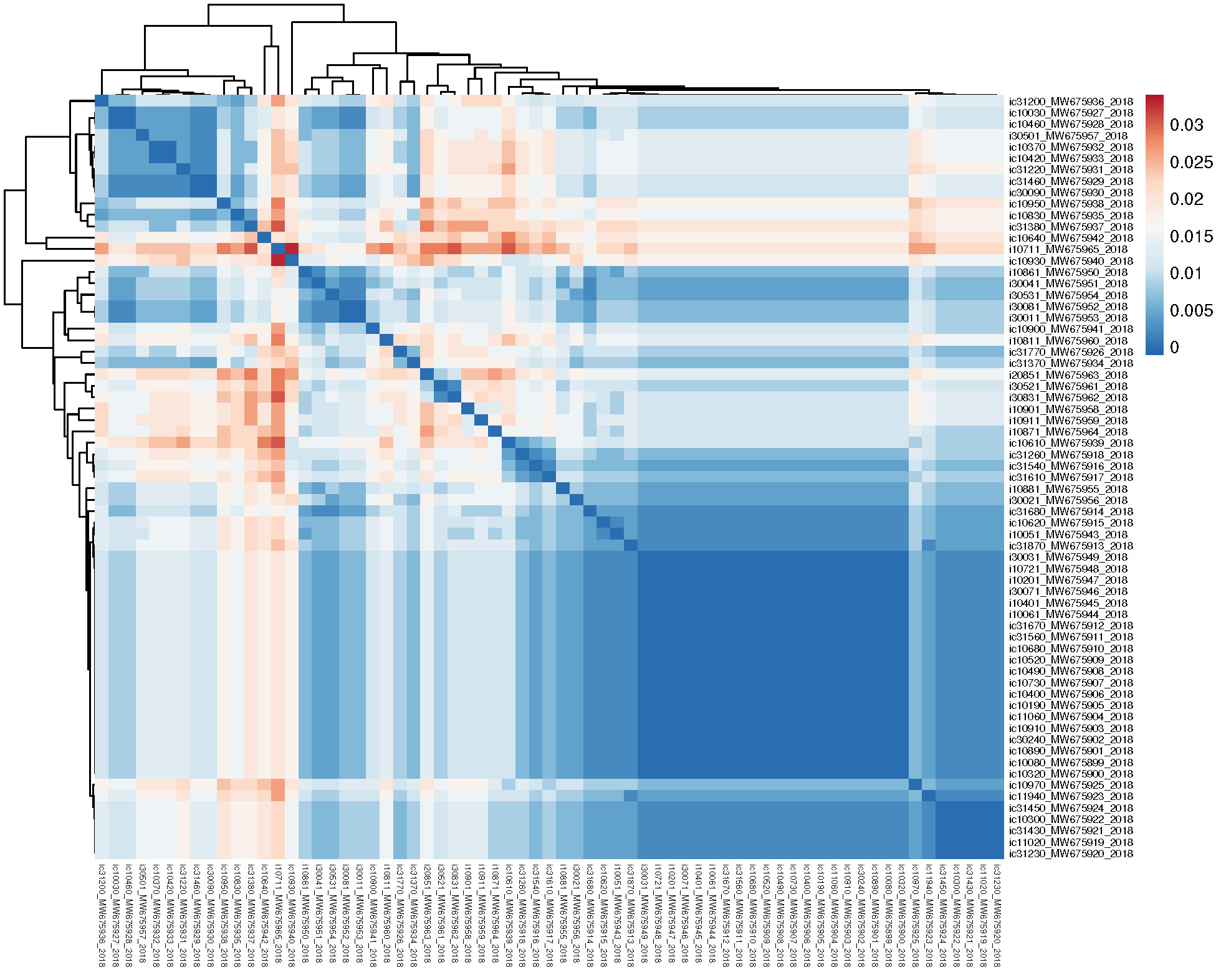

Supplement: S1 Fig — The color scale on the right depicts the color schemes applied for particular genetic distance thresholds. (TIF) [file pone.0317705.s001.tif]

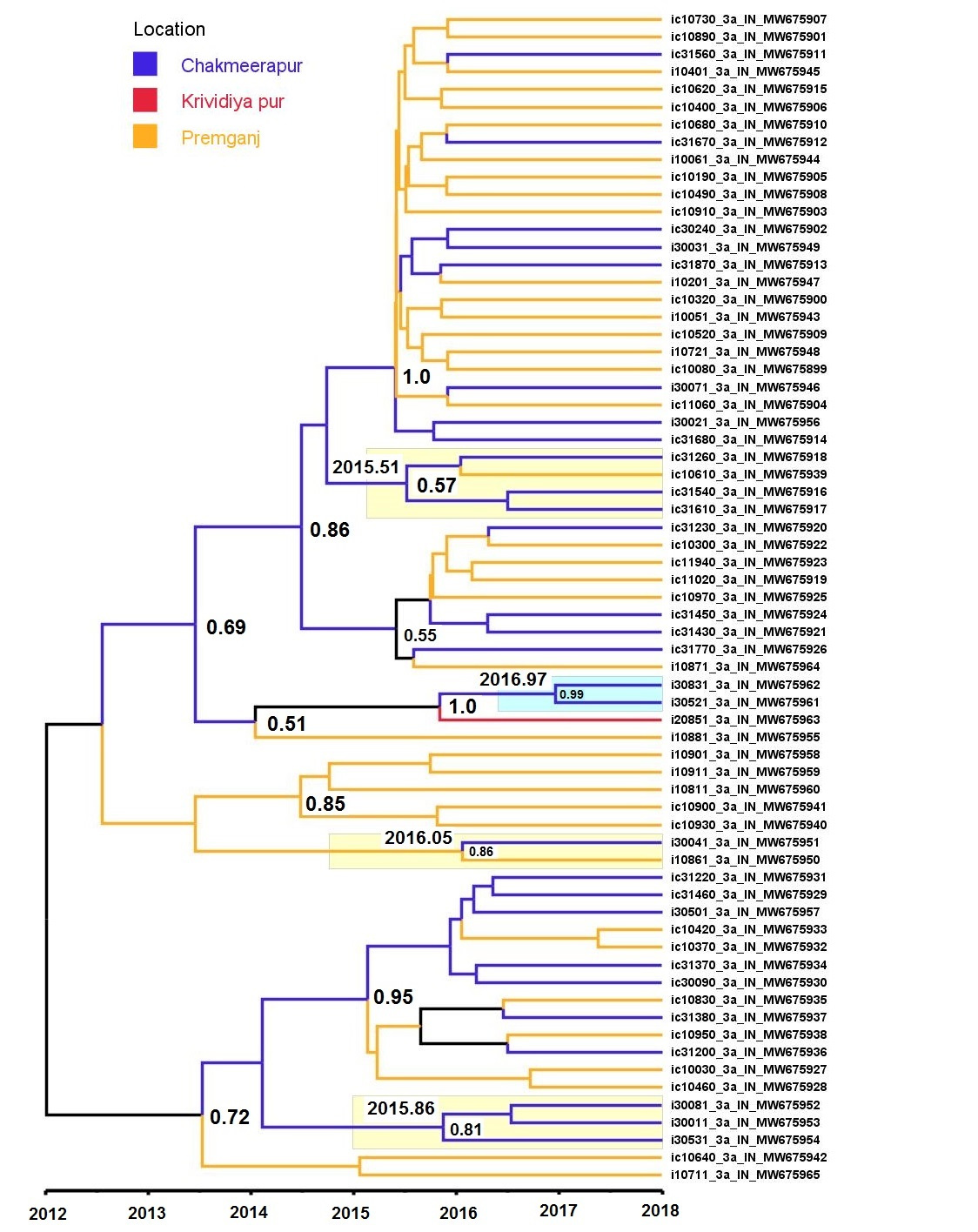

Supplement: S2 Fig — The legend on the left identifies the colors used to represent the individual locations. The figures at individual nodes represents the posterior probability. (TIF) [file pone.0317705.s002.tif]
